# Development and Psychometric Validation of the Mistreatment Questionnaire for Medical Trainees (MQMT) in Mexican Medical Students, Interns, and Residents

**DOI:** 10.3390/healthcare14193333

**Published:** 2026-10-06

**Authors:** Ramsés Barrera-Águila, Luis Eduardo Del Moral-Trinidad, Blanca Estela Ríos-González, Daniela De-Loza-Cázares, Perla Jazmín Figueroa-Martínez, Ingrid Patricia Dávalos-Rodríguez, Aniel Jessica Leticia Brambila-Tapia

**Affiliations:** 1 Doctorado en Farmacología, Departamento de Fisiología, Centro Universitario de Ciencias de la Salud (CUCS), Universidad de Guadalajara, Guadalajara 44340, Jalisco, Mexico; ramses.barrera7991@alumnos.udg.mx; 2 Doctorado en Ciencias de la Salud Pública, Departamento de Salud Pública, Centro Universitario de Ciencias de la Salud (CUCS), Universidad de Guadalajara, Guadalajara 44340, Jalisco, Mexico; luis.delmoral8315@alumnos.udg.mx; 3 Unidad Médico Familiar #92, Instituto Mexicano del Seguro Social (IMSS), Guadalajara 44990, Jalisco, Mexico; 4 Licenciatura en Medicina, Centro Universitario de Ciencias de la Salud (CUCS), Universidad de Guadalajara, Guadalajara 44340, Jalisco, Mexico; 5 Licenciatura en Enfermería, Centro Universitario de Ciencias de la Salud (CUCS), Universidad de Guadalajara, Guadalajara 44340, Jalisco, Mexico; 6 División de Genética, Centro de Investigación Biomédica de Occidente (CIBO), Instituto Mexicano del Seguro Social (IMSS), Guadalajara 44340, Jalisco, Mexico; 7 Departamento de Biología Molecular y Genómica, Centro Universitario de Ciencias de la Salud (CUCS), Universidad de Guadalajara, Guadalajara 44340, Jalisco, Mexico; 8 Departamento de Psicología Básica, Centro Universitario de Ciencias de la Salud (CUCS), Universidad de Guadalajara, Guadalajara 44340, Jalisco, Mexico

**Keywords:** mistreatment, medical training, medical students, medical trainees, residents, psychological mistreatment, academic mistreatment, physical mistreatment, discrimination, sexual harassment, confirmatory factor analysis, validation

## Abstract

**Introduction:** Mistreatment in medical training is a highly prevalent phenomenon that occurs in different types and forms. However, despite its high frequency, no measurement instrument has been validated that represents these different types of mistreatment in its factorial structure. Therefore, the objective of this study was to design the Mistreatment Questionnaire for Medical Trainees (MQMT) and to obtain initial evidence of its validity and reliability. **Methods:** Medical trainees at different stages (non-clinical and clinical undergraduate medical students, interns, and residents) were invited to participate through social media and personal invitation. The electronic survey included a 28-item pool adapted from previous mistreatment questionnaires, together with measures of anxiety (GAD-7), depression (PHQ-9), and perceived stress (PSS-10). The sample was randomly split into exploratory and confirmatory subsamples. **Results:** A total of 555 medical trainees were included (64% women; mean age, 23.7 years). Exploratory factor analysis (EFA; n = 280) suggested four factors, one of which combined physical mistreatment and discrimination items. A 16-item, five-factor model (academic mistreatment, psychological mistreatment, physical mistreatment, discrimination, and sexual harassment) was specified on empirical and theoretical grounds and compared with competing models in an independent subsample (n = 275), using weighted least squares with mean- and variance-adjusted test statistics (WLSMV) on polychoric correlations as the primary estimator and robust maximum likelihood (MLR) as a sensitivity analysis. The five-factor model showed good fit (CFI = 0.984, TLI = 0.980, RMSEA = 0.066, SRMR = 0.049); the MLR solution was consistent and favored the same model. Standardized loadings ranged from 0.814 to 0.980, and subscale reliability was satisfactory in the confirmatory subsample (α = 0.866–0.943; ω = 0.868–0.943). Academic mistreatment was the most frequently reported dimension, followed by psychological mistreatment. MQMT scores showed small-to-moderate positive correlations with symptoms of anxiety, depression, and stress, with stronger associations for academic and psychological mistreatment. **Conclusions:** The MQMT is a brief 16-item, five-dimensional instrument with initial evidence of validity and reliability in Mexican medical trainees. Subscale scores are the primary scores of the instrument, and the empirical distinction between discrimination with physical mistreatment and sexual harassment is not yet firmly established. Replication in other populations is needed.

## 1. Introduction

Mistreatment can be defined as ‘any act or omission that results in harm or risk of harm to a person’s health or well-being’ [1]. It is a widely documented phenomenon in educational and clinical settings in medical training, both among undergraduate and postgraduate students, to such an extent that it often seems inherent to medical culture [2,3,4]. Although it can be exercised by peers, it takes on the character of abuse when perpetrated by teachers or other figures with greater hierarchical power [1,5].

Mistreatment within the medical environment is usually classified into four main categories: physical, psychological, sexual, and academic. Physical mistreatment involves any action that causes harm using physical force. Psychological mistreatment includes emotional or social harm through verbal or non-verbal behavior, intimidation, discrimination, or abuse of power. Sexual harassment refers to acts that violate a person’s sexual integrity. Academic mistreatment includes actions that impair educational performance through ethically questionable practices, often carried out by teachers or educational authorities [1,6,7]. Discrimination is increasingly recognized as a specific type of mistreatment within medical education rather than merely a demographic characteristic associated with abusive experiences. It encompasses unfair or unequal treatment based on personal attributes such as race, ethnicity, gender, disability, sexual orientation, or other characteristics, and has been consistently associated with adverse educational experiences, psychological distress, burnout, and impaired professional development among medical trainees [4]. Discrimination may represent a distinct dimension of mistreatment because it reflects systematic inequities embedded within institutional and interpersonal interactions, differing from other forms of abuse such as psychological intimidation, academic mistreatment, or sexual harassment. Consequently, it has been proposed that discrimination be evaluated as an independent domain when assessing learning environments and trainee well-being [6].

The occurrence of mistreatment is related to multiple factors, including individual characteristics of the perpetrator and the victim, previous experiences of mistreatment, and relational dynamics within the clinical setting [7]. In medicine, the hierarchical structure is particularly relevant, as the tiered organization and distribution of power facilitate the emergence of abusive behavior, allowing mistreatment to become normalized as part of training practice [1,5]. Thus, both the work environment and institutional culture function as predisposing factors that favor its perpetuation [3,4].

The literature documents significant effects on the mental health of students exposed to mistreatment, including depressive symptoms, anxiety, increased stress, decreased self-esteem, and reduced self-confidence [3]. These alterations can lead to maladaptive coping strategies, such as substance or alcohol use, also affecting interpersonal relationships [7].

In clinical settings, mistreatment during training is associated with decreased empathy toward sick people and deficiencies in the doctor-patient relationship. At the academic level, it has been linked to lower academic performance, absenteeism, dissatisfaction with the career, and even dropping out of school [3,8].

Numerous international studies show the high prevalence of mistreatment in medical training. A systematic review and meta-analysis reported that approximately 60% of students and medical trainees have experienced some form of harassment or discrimination during their training. Several subsequent studies have shown that this phenomenon persists [4]. A similar study estimated an overall prevalence of 51% of bullying among residents in a meta-analysis. In specific specialties, the prevalences may be higher [5]. A study of 7409 participants reported a prevalence of mistreatment of more than 50% among general surgery residents in the United States, ranging from 10.3% to 66.7% depending on the type of surgical specialty [9]. Similarly, a study documented a 100% prevalence among cardiology residents in Argentina, with the main cause of mistreatment being criticism for not performing administrative tasks [10].

A review of undergraduate students reported ranges between 11% and over 86.1%, depending on the educational context and measurement methods [8]. In Latin America and Mexico, the problem is also widely recognized. For example, a Mexican study found prevalences of up to 84% among residents of different specialties [11]. The lowest percentages were found among family medicine residents, observed in 15% in another Mexican study [12].

Mistreatment is a complex construct that requires clear and understandable definitions for those involved in assessments. Although there are various scales that seek to measure it, no instrument with a validated factorial structure has been identified for medical trainees. One of the most widely used instruments for assessing mistreatment in medical training is the Workplace Bullying in Junior Doctors Questionnaire (WBJDQ), developed by Quine, which consists of 21 items that assess specific behaviors of abuse, intimidation, humiliation, exclusion, work overload, and threats within clinical contexts [13]. Since its creation, the WBJDQ has been adapted and used in various countries, including Ireland, Pakistan, Colombia, and Malaysia [14,15,16,17].

However, it is important to note that the original publication did not report a formal exploratory or confirmatory factor analysis, nor did it present empirical evidence on the dimensional structure of the questionnaire or the grouping of items into subscales representing the different types of mistreatment it addresses. In most subsequent adaptations, the instrument has been used globally or with conceptual groupings derived from the literature, without systematically evaluating its factorial structure through exploratory or confirmatory analyses.

To our knowledge, only one postgraduate thesis conducted in Colombia has explored the factorial structure of the WBJDQ and its subscales associated with different types of mistreatment; however, this study did not perform a confirmatory factor analysis, and its results have not been published in a peer-reviewed journal, which limits its dissemination, replicability, and use as a methodological reference [18]. Because of this, we decided to measure the instrument along with other additional mistreatment items in a sample of Mexican medical trainees, with the aim of developing a new and improved instrument to measure mistreatment in medical personnel in training both in Mexico and in other countries. Therefore, the objective of the study was to design an instrument to measure mistreatment in medical trainees and to obtain initial evidence of its validity and reliability.

## 2. Methods

### 2.1. Design

A cross-sectional instrumental validation study was conducted, aimed at cultural adaptation, item reduction, and evaluation of the psychometric properties of a questionnaire on mistreatment among medical trainees (undergraduate medical students, interns, and residents) in Mexico. The study was divided into two phases: an exploratory factor analysis (EFA) phase (n = 280) and a confirmatory factor analysis (CFA) phase (n = 275), using independent samples. The complete sample was randomly split into two subsets of similar size using computer-generated random allocation in R (version 4.5.1) [19] through the RStudio interface (version 2026.09.0) before the factor analyses were performed.

### 2.2. Subjects

The target population consisted of medical students and medical trainees in Mexico with the following selection criteria: (a) be an active student in the field of medicine or a medical specialty, (b) be studying at an institution in Mexico, and (c) be over 18 years of age.

The exclusion criterion was (a) not completing the measurement of the instruments.

### 2.3. Sample Size Calculation

For the EFA, a minimum of 10 participants per item has been recommended [20]; with 28 initial items, a minimum of 280 participants was required. For the CFA, rules of thumb based on the ratio of participants to freely estimated parameters [21] are difficult to apply to the present model, because categorical estimation additionally requires estimating the item thresholds (16 items × 4 thresholds = 64 parameters) together with the loadings, factor correlations, and residual terms; under that criterion, a subsample of 275 participants would not reach the recommended lower bound. The adequacy of the confirmatory subsample was therefore judged against simulation evidence on the WLSMV estimator, which indicates that valid results (adequate χ^2^ approximation, unbiased parameter estimates, and appropriate standard errors) are obtained with at least 250 observations for models of reasonable complexity (2–4 items per factor) and with 500 observations for more complex models (5–6 items per factor) [22]. Given that the present model involves a moderately complex structure with high standardized loadings (all ≥ 0.81), a subsample of 275 participants falls within the range supported by this simulation evidence.

### 2.4. Procedure

Non-probabilistic snowball sampling was used for recruitment. This method was selected because the target population (medical students and trainees) is a specialized, hard-to-reach group distributed across multiple institutions in Mexico. The research team distributed an electronic questionnaire through social networks such as WhatsApp, Facebook, and Instagram. Medical trainees were also invited to participate in the study directly by the research team. This procedure made it possible to reach mainly young people enrolled in undergraduate and graduate programs in health sciences. The sociodemographic data collected included gender, age, type of training (non-clinical undergraduate, clinical undergraduate, interns, and residents), whether they were working, and socioeconomic status classified from very low to very high.

### 2.5. MQMT Instrument Design

The questionnaire was based on the original version of the WBJDQ; one original item (discrimination on grounds of race and gender) was divided into two independent items, one for each type of discrimination. This version was translated and adapted into Spanish by the research team. Subsequently, the instrument was revised with the aim of incorporating additional items that would allow for a more accurate assessment of the different types of mistreatment in the medical education and training context. All the items included underwent a process of translation and back-translation, followed by cultural adaptation for the Mexican population, in which the research team proposed conceptual and linguistic adjustments. Translation and cultural adaptation followed the stages recommended by Beaton et al. [23]. Forward translation from English into Spanish was performed independently by two bilingual translators, both trained in medicine, and one of them with experience in medical education. Discrepancies were resolved by consensus in a meeting of the research team, and the reconciled version was back-translated into English by a bilingual translator. The back-translation was compared with the original items to confirm conceptual equivalence. The items taken from the questionnaires of Derive et al. [11] and Reyes-Arellano et al. [7] were originally written in Spanish and had previously been applied to Mexican residents and undergraduate medical students, respectively, which supports their linguistic and cultural relevance for the target population. The pre-final 28-item version was reviewed by five experts in medical education, psychology, and psychometrics, who belong to the research team and who reviewed the relevance and clarity of each item qualitatively [24], and it was pilot-tested with ten medical trainees from different training stages, who were asked about the comprehensibility of each item. The initial item pool was intended to cover the five domains of mistreatment described in the literature (academic, psychological, and physical mistreatment, discrimination, and sexual harassment) [1,4,6,7]; the intended domain of each of the 28 items is reported in Supplementary Table S1.

The initial item pool consisted of 28 items, 22 of which came from the original version of the WBJDQ instrument. In addition, three items were incorporated from the mistreatment questionnaire proposed by Derive et al., applied to medical residents in Mexico, which explores sexual harassment and discrimination [11]. Three items were also added from the instrument on mistreatment in preclinical students in Mexico developed by Reyes-Arellano et al., also on discrimination based on sexual orientation and sexual harassment [7]. The complete instrument used can be found in Table 1.

In the original version of the WBJDQ, the items assessed the presence of specific abusive behaviors experienced during the last 12 months. In a subsequent adaptation to Spanish, these items have been operationalized using Likert-type frequency scales, commonly ranging from 1 to 4 [18]. In the present study, respondents were asked about these behaviors experienced during their training as physicians. In addition, a five-point Likert scale was used, with values ranging from 0 to 4 (0 = never, 1 = almost never, 2 = sometimes, 3 = almost always, 4 = always). This decision was based on two methodological considerations: (a) the explicit inclusion of the category “never” with a value of zero allows for a more intuitive interpretation of the absence of the phenomenon and avoids overestimating the frequency of mistreatment, and (b) the use of a five-point scale increases sensitivity to detect frequency gradients in experiences of mistreatment, which is especially relevant in training contexts where these behaviors may occur intermittently.

### 2.6. Additional Psychological Instruments

To obtain evidence of validity based on relationships with theoretically related variables, three measures previously validated in Spanish-speaking populations were used: stress level, assessed with the Perceived Stress Scale (PSS-10; item range 0–4) [25]; depressive symptoms, measured with the Patient Health Questionnaire-9 (PHQ-9; item range 0–3) [26,27]; and anxiety symptoms, assessed with the Generalized Anxiety Disorder-7 (GAD-7; item range 0–3) [28].

### 2.7. Statistical Analysis

Qualitative variables were described using frequencies and percentages, while quantitative variables were expressed using means and standard deviations. For ordinal items, response distributions (frequencies per category) were also examined.

EFA was conducted on a sample of 280 participants. Prior to EFA, sample adequacy was assessed using the Kaiser–Meyer–Olkin (KMO) index (acceptable threshold ≥ 0.70) and Bartlett’s test of sphericity (*p* < 0.05) [29]. Factor extraction was performed using the unweighted least squares method on the correlation matrix with Promax oblique rotation, assuming correlation between factors. Factor retention was determined using parallel analysis, complemented by inspection of the scree plot and theoretical interpretability. Factor loadings ≥ 0.40 were considered acceptable for item retention [20]. Items with cross-loadings (difference < 0.15 between two factors) were considered for removal. Item reduction was performed entirely in the EFA subsample, according to the following criteria: (a) primary loading < 0.40; (b) cross-loading as defined above; (c) loading on a factor inconsistent with the conceptual content of the item; and (d) semantic redundancy with another item of the same factor, with the aim of obtaining a brief instrument with non-redundant indicators covering each dimension. All item decisions in the final version were taken in the EFA subsample. The loadings, communalities, decision, reason, and stage of decision for each of the 28 items are reported in Supplementary Table S1.

CFA was conducted on the independent subsample of 275 participants. Four competing models were specified: M1, a one-factor model; M2, a four-factor model reproducing the EFA solution, in which the physical mistreatment and discrimination items loaded on a single factor; M3, the theoretically specified model with five correlated factors; and M4, a second-order model in which the five first-order factors loaded on a general mistreatment factor. Weighted least squares with mean- and variance-adjusted test statistics (WLSMV) on the matrix of polychoric correlations was used as the primary estimator. This choice was based on the measurement characteristics of the instrument: the items are ordinal, and several low frequency types of mistreatments (physical mistreatment, discrimination, and sexual harassment) showed marked floor effects and strongly asymmetric category thresholds. Under these conditions, categorical estimators that model the underlying continuous response variable through polychoric correlations provide less biased factor loadings and more accurate parameter estimates than normal-theory estimators, which are particularly sensitive to asymmetric thresholds and tend to underestimate large loadings [30,31,32]. Because the WLSMV test statistic is not a likelihood-based statistic and does not yield information criteria, robust maximum likelihood (MLR) was used as a sensitivity analysis for the same four models; MLR also allowed the models to be compared through the Akaike (AIC) and Bayesian (BIC) information criteria, with lower values indicating a better balance between fit and parsimony [32,33]. Model fit was evaluated using the robust comparative fit index (CFI), Tucker–Lewis index (TLI), standardized root mean square residual (SRMR), and root mean square error of approximation (RMSEA) with its 90% confidence interval. Values ≥ 0.90 for CFI and TLI and ≤ 0.08 for RMSEA and SRMR were considered indicative of acceptable model fit, although these thresholds were interpreted alongside theoretical considerations and the empirical characteristics of the data [34]. Nested models (M2, M4, and M1, each compared with M3) were compared using scaled χ^2^ difference tests—the scaled-and-shifted test for WLSMV [35] and the Satorra–Bentler test for MLR [36]. Three additional models, each constraining the correlation between a pair of highly correlated factors (academic–psychological, physical–discrimination, and discrimination–sexual harassment), were also compared with M3. Under WLSMV, the constraint was imposed directly on the latent correlation, which was fixed at 1 (Δdf = 1). Under MLR, these models did not converge, so the two factors were merged into a single factor instead (Δdf = 4); this specification is more restrictive, because it additionally equates their correlations with the remaining factors. Every difference test and every ΔCFI value is therefore reported together with the estimator from which it was obtained. Discriminant validity between factors was examined from the upper limit of the 95% confidence interval of each latent correlation [37] and from the heterotrait–monotrait ratio of correlations (HTMT) computed on the polychoric correlation matrix, using 0.85 and 0.90 as reference thresholds [38].

For descriptive purposes only, the mean score of each dimension was grouped into three frequency bands anchored to the response labels: <1 (between “never” and “almost never”; infrequent), 1 to <2 (between “almost never” and “sometimes”; occasional), and ≥2 (“sometimes” or more often; recurrent). These study-specific bands are not validated cut-off points and were not intended for screening or for defining clinically or institutionally relevant severity levels.

Evidence of validity based on relationships with other variables (concurrent evidence within the expected nomological network) was examined using Spearman’s correlations, owing to the non-normal distribution of the data, between MQMT dimension scores and measures of stress (PSS-10), depression (PHQ-9), and anxiety (GAD-7) [24]. Because these instruments assess psychological discomfort rather than mistreatment, positive, small-to-moderate correlations were expected. Internal consistency was assessed for each subscale using Cronbach’s alpha and McDonald’s omega, considering values ≥ 0.70 as acceptable [39,40]. Because the measurement model was tested in the CFA subsample, reliability is reported for that subsample and, for comparison, for the EFA subsample. Consistent with the categorical treatment of the items, ordinal alpha based on polychoric correlations and model-based (categorical) omega derived from the WLSMV solution were also calculated, together with the Spearman–Brown coefficient for the two-item physical mistreatment subscale [41]. Descriptive analyses and the exploratory factor analysis were performed using the JASP program, version 0.95.3 [42], and the confirmatory models, reliability coefficients, and model comparisons were estimated in R (version 4.5.1) [19] using the lavaan (verion 0.7.2) [43], semTools (version 0.5-9) [44], and psych (version 2.6.5) [45] packages.

### 2.8. Ethical Considerations

The study was approved by the Ethics and Research Committee of the Health Sciences University Center from the University of Guadalajara (registration number: CI-01525). The questionnaire was anonymous and did not ask for any identifying information. All participants gave their informed consent at the beginning of the electronic questionnaire. The study was conducted in accordance with the ethical principles of The Declaration of Helsinki.

## 3. Results

A total of 555 medical trainees were included and randomly allocated to two independent subsamples: 280 for the EFA and 275 for the CFA. The descriptive statistics data presented below correspond to the combined sample (N = 555) because the two samples did not show significant differences in demographic characteristics.

The mean age was 23.7 ± 4.6 years. Of the total, 355 (64%) were women. In terms of training level, 43.6% were non-clinical undergraduate students, 16.6% clinical undergraduate students, 8.3% interns, and 31.5% residents. Regarding sociodemographic characteristics, 48.8% of participants reported having a partner, 3.4% had children, and 18.7% reported having a job in addition to their studies. Regarding socioeconomic status, the highest proportion (26.3%) considered it to be very high (Table 2).

The EFA was performed on the 28 items with the EFA sample (n = 280). The KMO measure of sampling adequacy was 0.944, and Bartlett’s test of sphericity was significant (χ^2^ = 7462.7, df = 378, *p* < 0.001), indicating good adequacy of the data for factor analysis.

Parallel analysis suggested the retention of four factors (Table 3), which was consistent with the scree plot. In this solution, one factor combined the physical mistreatment items (items 10 and 11) with the discrimination items (items 22, 23, and 26) and one item on withholding information (item 12), whereas the gender discrimination item (item 25) loaded on the sexual harassment factor. Because physical mistreatment and discrimination represent conceptually distinct types of mistreatment, a five-factor structure was hypothesized for the confirmatory phase, and its fit was formally compared with that of the four-factor EFA-derived structure in the CFA subsample.

Item reduction followed the criteria described in Section 2.7, and the decision, reason, and stage of decision for each of the 28 items are detailed in Supplementary Table S1. Items 2, 4, and 14 were removed because of cross-loadings; items 12 and 25 were removed because they loaded on factors inconsistent with their content, the latter being particularly relevant because the gender discrimination item loaded on the sexual harassment factor rather than on the discrimination factor; and in seeking to have a brief yet representative questionnaire, items 1, 5, 9, 13, 19, 20, and 21 were removed because of semantic redundancy with retained items of the same factor that had higher factor loadings. Four items were retained for the academic and psychological mistreatment dimensions, three for discrimination, three for sexual harassment, and two for physical mistreatment. The resulting instrument comprised 16 items distributed across five dimensions: academic mistreatment, psychological mistreatment, physical mistreatment, discrimination, and sexual harassment (Table 4). The final version of the scale was named the Mistreatment Questionnaire for Medical Trainees (MQMT).

To examine the response patterns of the 16 retained items, frequency distributions were calculated in the EFA subsample for each item across the five response categories (0 = never to 4 = always). Table 5 presents the number and percentage of participants selecting each response option for every item, organized by the five dimensions of the MQMT; marked floor effects were observed for the physical mistreatment, discrimination, and sexual harassment items, which motivated the categorical estimation strategy described in Section 2.7.

The refined 16-item version was used for all confirmatory analyses. Several competing factor structures were specified and compared in the CFA subsample, including one-, four-, five-, and second-order factor models, as well as a model constraining the correlation between physical mistreatment and discrimination to unity. The fit of all competing models under both estimators is shown in Table 6, and the corresponding nested comparisons are detailed in Supplementary Tables S3 and S4. Under the primary estimator (WLSMV), the five-factor model (M3) fitted better than the one-factor model (scaled-and-shifted Δχ^2^ = 176.03, Δdf = 10, *p* < 0.001; ΔCFI = −0.050), the four-factor EFA-derived model (Δχ^2^ = 22.48, Δdf = 4, *p* < 0.001; ΔCFI = −0.003), and the second-order model (Δχ^2^ = 66.70, Δdf = 5, *p* < 0.001; ΔCFI = −0.018). The MLR sensitivity analysis reproduced this ordering with larger differences in approximate fit and clearly favored M3 on the information criteria (vs. M1: Satorra–Bentler Δχ^2^ = 253.61, Δdf = 10, *p* < 0.001, ΔCFI = −0.248, ΔAIC = 882.2, ΔBIC = 846.0; vs. M2: Δχ^2^ = 58.10, Δdf = 4, *p* < 0.001, ΔCFI = −0.046, ΔAIC = 164.8, ΔBIC = 150.3; vs. M4: Δχ^2^ = 66.32, Δdf = 5, *p* < 0.001, ΔCFI = −0.026, ΔAIC = 89.3, ΔBIC = 71.2). It should be noted that, under WLSMV, the difference in approximate fit between M2 and M3 was small (ΔCFI = 0.003); the preference for the five-factor model therefore rests on the convergence of the difference tests under both estimators and on the information criteria of the MLR solution rather than on a large difference in global fit.

The five-factor correlated model (M3) was retained as the final model based on its overall fit and theoretical interpretability (Table 6). Using WLSMV, M3 showed good fit (CFI = 0.984, TLI = 0.980, RMSEA = 0.066, 90% CI [0.054, 0.078], SRMR = 0.049). As a sensitivity analysis, the same model was estimated using robust maximum likelihood (MLR), yielding a similar pattern of fit (CFI = 0.969, TLI = 0.961, RMSEA = 0.065, SRMR = 0.048). When fitted to the EFA subsample, the final five-factor model also showed good fit (CFI = 0.990, TLI = 0.988, RMSEA = 0.060, SRMR = 0.053), providing evidence for the stability of the factor structure across the independent subsamples.

Fully standardized factor loadings are reported in Table 7. All items showed high and statistically significant loadings on their respective factors, ranging from 0.814 to 0.980 in the WLSMV solution. Internal consistency was satisfactory across all five dimensions in the CFA subsample, in which the measurement model was tested, with Cronbach’s α ranging from 0.866 to 0.943 and McDonald’s ω from 0.868 to 0.943. Consistent with the categorical estimation strategy, ordinal α ranged from 0.907 to 0.975 and model-based (categorical) ω from 0.873 to 0.922. Values were comparable in the EFA subsample (α = 0.768–0.932; ω = 0.790–0.932), where the discrimination subscale showed the lowest estimate. Given that the physical mistreatment dimension comprised only two items, its reliability was additionally assessed using the Spearman–Brown coefficient, which was 0.917 in the CFA subsample and 0.915 in the EFA subsample.

Table 8 shows the standardized latent correlations between the factors with their 95% confidence intervals. All correlations were positive and statistically significant and ranged from 0.614 (academic mistreatment with physical mistreatment) to 0.943 (discrimination with sexual harassment). Three pairs of factors showed correlations high enough to raise concerns about discriminant validity: academic and psychological mistreatment correlated 0.852 (95% CI: 0.804–0.901; ~73% shared latent variance; HTMT = 0.841), physical mistreatment and discrimination correlated 0.890 (95% CI: 0.833–0.950; ~79% shared variance; HTMT = 0.894), and discrimination and sexual harassment correlated 0.943 (95% CI: 0.911–0.975; ~89% shared variance; HTMT = 0.943). All remaining HTMT values were ≤ 0.791. These three pairs therefore reached or exceeded the thresholds commonly used to indicate a lack of discriminant validity, whether judged by the upper limit of the 95% confidence interval of the latent correlation or by the HTMT ratio. To evaluate whether these dimensions were empirically distinguishable, models fixing each of these correlations to 1 were compared with M3 under WLSMV. Merging academic and psychological mistreatment produced a clear deterioration in fit (scaled-and-shifted Δχ^2^ = 41.14, Δdf = 1, *p* < 0.001; ΔCFI = −0.011), whereas merging physical mistreatment with discrimination (Δχ^2^ = 13.71, Δdf = 1, *p* < 0.001; ΔCFI = −0.004) or discrimination with sexual harassment (Δχ^2^ = 13.41, Δdf = 1, *p* < 0.001; ΔCFI = −0.001) produced statistically significant but negligible changes in approximate fit. Under MLR, where these models were specified by merging the two factors (Δdf = 4), the same pattern was observed (academic–psychological: Satorra–Bentler Δχ^2^ = 79.63, *p* < 0.001, ΔCFI = −0.036; discrimination–sexual harassment: Δχ^2^ = 16.99, *p* = 0.002, ΔCFI = −0.010). Because scaled difference tests are sensitive to trivial misfit at this sample size, these results indicate that the separation of academic and psychological mistreatment is empirically supported, whereas the distinction of discrimination from sexual harassment, and to a lesser extent from physical mistreatment, is not firmly established and currently rests mainly on content considerations. Detailed comparisons are reported in Supplementary Tables S3 and S4.

Based on the frequency of reported experiences, participants were grouped into three descriptive frequency bands for each dimension of the MQMT: infrequent, occasional, and recurrent (see Section 2.7). These bands are study-specific and do not represent validated severity levels. As shown in Table 9, the distribution differed markedly across dimensions. Academic mistreatment showed the highest proportion of participants in the occasional (25.4%) and recurrent (22.3%) bands, followed by psychological mistreatment (occasional: 29.9%; recurrent: 16.4%). In contrast, 84.9%, 84.0%, and 81.8% of participants were in the infrequent band for physical mistreatment, discrimination, and sexual harassment, respectively.

Cronbach’s alpha coefficient was 0.83 for the PSS-10, 0.93 for the GAD-7, and 0.90 for the PHQ-9, indicating good to excellent reliability in all cases. All MQMT dimensions showed statistically significant positive correlations with indicators of psychological discomfort; correlations were small to moderate for academic and psychological mistreatment (ρ = 0.31–0.37) and small for physical mistreatment, discrimination, and sexual harassment (ρ = 0.09–0.22) (Table 10).

## 4. Discussion

This study describes the design, cultural adaptation, and initial psychometric evaluation of the Mistreatment Questionnaire for Medical Trainees (MQMT) in a Mexican population of medical students, interns, and residents. The final instrument comprises 16 items distributed across five dimensions: academic mistreatment, psychological mistreatment, physical mistreatment, discrimination, and sexual harassment. The instrument showed satisfactory subscale reliability, a five-factor structure that was supported in an independent subsample and compared favorably with competing models, and theoretically expected associations with psychological discomfort.

The exploratory factor analysis suggested a four-factor solution, in which the indicators of physical mistreatment (physical violence and damage to personal property) loaded on the same factor as the discrimination items, despite these representing conceptually distinct types of mistreatment. Rather than adopting the exploratory solution without further testing, we specified a theoretically informed five-factor model and compared it directly with the four-factor EFA-derived model in an independent subsample. The five-factor model fitted significantly better with both estimators. It should nevertheless be acknowledged that, under the categorical estimator, the two models differed only marginally in approximate fit indices and that the four-factor model also showed acceptable fit, so that the advantage of the five-factor solution rests on the coincidence of the difference tests, the information criteria of the MLR solution, and the conceptual distinction between the two dimensions rather than on a large difference in global fit. The grouping observed in the EFA may partly reflect a shared distributional feature of these items—very low endorsement and strong floor effects (Table 5)—rather than conceptual similarity [46]. This approach is consistent with psychometric recommendations that consider exploratory factor analysis as a hypothesis-generating procedure and confirmatory factor analysis as a means of testing theoretically informed competing models [21,34].

Consequently, the confirmatory model retained five conceptually differentiated dimensions. This solution is consistent with previous literature describing mistreatment during medical training as a multidimensional phenomenon encompassing academic abuse, psychological abuse, physical aggression, discrimination, and sexual harassment rather than a single continuum of hostile behaviors [4,7,11]. The separation of physical mistreatment from discrimination improves the interpretability of the instrument and may facilitate the identification of specific types of mistreatment that require different institutional prevention strategies. The separation of discrimination from sexual harassment, however, received only weak empirical support: the two factors correlated 0.943, the HTMT ratio reached the same value, and merging them reduced the CFI by only 0.001. Their distinction therefore rests mainly on content, since both domains have different institutional and legal implications, and should be regarded as provisional until it is examined in samples with a higher endorsement of these behaviors.

In the case of the gender discrimination item (item 25), it loaded on the sexual harassment factor rather than on the discrimination factor in the EFA. This empirical pattern is itself informative, since gender-based discrimination and sexual harassment frequently co-occur and may be perceived by trainees as expressions of the same gendered hostility [47,48]; nevertheless, its removal was necessary to preserve the conceptual clarity of the discrimination dimension based on statistical criteria. As a consequence, the discrimination subscale covers discrimination based on race, on illness or disability, and on sexual orientation, but not gender-based discrimination, which is a content-validity limitation that future versions should address, for example by developing gender discrimination items that are clearly differentiated from sexual harassment behaviors [21,49].

The final version includes one dimension represented by only two indicators (physical mistreatment). Although the literature generally recommends a minimum of three to five indicators per factor for optimal model stability [21,50], factors with two indicators can be technically identified under specific conditions, such as when they are correlated with other factors in the model [21]. In the present study, this dimension showed very high standardized loadings (0.968 and 0.980) and adequate internal consistency (α = 0.933; Spearman–Brown = 0.916) [41], and it was retained because of its theoretical importance and conceptual distinctiveness from other types of mistreatment [4,11]. Nevertheless, this subscale should be regarded as preliminary: two-indicator factors are more vulnerable to estimation instability and sample-specific results, and their limited content restricts construct representation. Additional items on other forms of physical aggression should be developed and independently validated before this dimension is considered a stable subscale.

Confirmatory factor analysis was estimated with WLSMV on polychoric correlations. This decision follows from the measurement characteristics of the MQMT: the response format is ordinal and several dimensions showed marked floor effects and strongly asymmetric thresholds, conditions under which normal-theory estimators can underestimate large loadings and distort parameter estimates, whereas categorical estimators recover the associations among the underlying response variables more accurately [30,31,32]. Because the WLSMV statistic does not provide information criteria, MLR was used as a sensitivity analysis, which also allowed comparison of the competing models through AIC and BIC [33]. The MLR sensitivity analysis yielded the same model preference as the primary WLSMV analysis. Standardized loadings were generally higher under WLSMV than under MLR, which is consistent with differences in the treatment of ordinal and highly skewed response distributions between categorical and continuous-variable estimators. This difference is methodologically relevant: estimates obtained with normal-theory estimators in instruments with strong floor effects are likely to underestimate both the strength of the item–factor relationships and the degree of overlap between dimensions.

Academic and psychological mistreatment were strongly correlated (r = 0.852, 95% CI: 0.804–0.901), sharing approximately 73% of their latent variance, and some items, of the original 28-item pool, cross-loaded on both factors in the EFA (items 2 and 4). This finding deserves critical consideration. In hierarchical training environments, abusive educational practices—such as excessive demands, unfair evaluation, or the undervaluing of trainees’ efforts—are frequently enacted through humiliation, sarcasm, and intimidation, a pattern described as “teaching by humiliation” [51], and excessive academic demands often co-occur with verbal abuse [4,52]. Therefore, academic and psychological mistreatment are not independent experiences in practice and may be partly different expressions of the same abusive hierarchical relationship. At the same time, the two dimensions have different item content: academic mistreatment captures the misuse of educational and supervisory authority over trainees’ work (e.g., impossible deadlines or shifting responsibilities without consultation), whereas psychological mistreatment captures direct interpersonal aggression (e.g., humiliation, threats, or degrading jokes). We retained both dimensions separately because they correspond to distinct practices with different targets for institutional intervention and because merging them could reduce the visibility of academic mistreatment, which is frequently normalized as a legitimate part of training. The second-order model, which represents these dimensions as facets of a general mistreatment factor, fitted worse than the model with five correlated factors. Future studies using bifactor models [53], multitrait–multimethod designs, or qualitative approaches should examine whether trainees themselves perceive these experiences as distinct.

Academic mistreatment was the most frequently reported form of abuse, followed by psychological mistreatment. These findings are consistent with previous international studies demonstrating that excessive workload, unrealistic expectations, humiliation, and verbal abuse have become normalized within many medical training environments [4,7,11]. Conversely, physical mistreatment, discrimination and sexual harassment showed considerably lower frequencies. Although these types of mistreatment were less commonly reported, previous evidence suggests that they are frequently underreported because of stigma, fear of retaliation, and normalization within hierarchical institutions [54]. Their inclusion as independent dimensions therefore remains clinically and ethically relevant given their documented association with psychological distress and adverse educational outcomes.

The associations between MQMT scores and symptoms of stress, anxiety, and depression provide evidence of validity based on relationships with theoretically related variables rather than convergent validity in the strict sense, because these instruments assess psychological discomfort, a potential correlate or consequence of mistreatment, and not mistreatment itself [24]. Correlations were small to moderate, as expected for distinct constructs, and stronger for academic and psychological mistreatment, consistent with previous research demonstrating that persistent exposure to hostile educational environments negatively affects emotional well-being, professional identity, and academic performance among medical trainees [8,55]. The weaker associations observed for physical mistreatment, discrimination, and sexual harassment may partly reflect restricted variance due to their low frequency, although they may also reflect genuine differences in their association with psychological discomfort; the present design does not allow these explanations to be distinguished. Future studies should obtain evidence of convergent validity with other mistreatment measures and of discriminant validity with theoretically unrelated constructs.

The present study has potential implications for both research and institutional practice. The MQMT provides a brief multidimensional instrument that may allow a standardized assessment of different types of mistreatment across medical schools and residency programs. Once its measurement properties have been replicated, it could be used to monitor learning environments and to evaluate institutional interventions—an area in which the published evidence is still scarce and of limited methodological quality [56]—and to identify specific domains requiring preventive action, thereby contributing to safer and healthier educational settings [57]. Future studies should examine whether mistreatment during clinical training may also be associated with missed or delayed care, extending its potential consequences from trainee well-being to care delivery [58]; this hypothesis is supported by evidence that work environment characteristics are associated with the quality of care [58,59] and that exposure to rudeness impairs the diagnostic and procedural performance of medical teams [60].

This study should be interpreted considering several methodological limitations. First, data were collected via self-report, which may be affected by social desirability bias and may underestimate the prevalence of certain types of mistreatment. Nonetheless, the anonymity of the survey likely mitigated this concern. Second, although the sample size was adequate for the factor analyses conducted, the use of non-probabilistic snowball sampling may limit the generalizability of the findings to other populations or institutional contexts. Perceptions and expressions of mistreatment may vary across regions, types of institutions (public vs. private), educational levels, and cultural norms. Third, the latent correlations between several dimensions were very high, particularly between discrimination and sexual harassment (0.943; HTMT = 0.943) and between physical mistreatment and discrimination (0.890; HTMT = 0.894). Although the models merging these factors fitted significantly worse, the corresponding changes in approximate fit were negligible (ΔCFI = −0.001 and −0.004), so the empirical distinction between these dimensions cannot be considered firmly established. This overlap most likely reflects the joint occurrence of these low-frequency experiences in the same trainees; it should be re-examined in samples with a higher endorsement of these behaviors and, if it persists, a combined dimension should be considered. Fourth, the sample was heterogeneous in training stage (43.6% non-clinical undergraduate students and 31.5% residents), and experiences and interpretations of academic pressure, workplace violence, discrimination, and harassment may differ between preclinical students and trainees working in clinical settings. Fifth, although the items were translated and back-translated and several items came from instruments previously used in Mexican medical trainees, no formal quantitative content validity index was obtained from an independent expert panel; moreover, the removal of the gender discrimination item limits the content coverage of the discrimination dimension. Sixth, the physical mistreatment dimension was represented by only two items and should be considered preliminary; future research should develop additional items to assess this construct more comprehensively. Seventh, the number of parameters estimated under categorical estimation, which includes the thresholds of every item, is large relative to the size of the CFA subsample, so the estimates of the least endorsed items should be regarded as provisional. Finally, the frequency bands used to describe the results are descriptive categories developed for this study and not validated cut-off points; criterion-referenced thresholds for screening or institutional decision-making should be established in future research using external criteria.

## 5. Conclusions

The MQMT is a brief 16-item questionnaire that assesses five dimensions of mistreatment in medical training: academic mistreatment, psychological mistreatment, physical mistreatment, discrimination, and sexual harassment. In Mexican medical trainees, the five-factor structure was supported in an independent subsample using an estimator appropriate for ordinal items with floor effects, which was preferred over the four-factor EFA-derived and second-order models, and was replicated in the exploratory subsample; subscale reliability was satisfactory and scores were associated with psychological discomfort in the expected direction. Subscale scores should be considered the primary scores of the instrument. The present evidence does not support the use of an overall composite score, because the second-order model fitted worse than the model with five correlated factors and no bifactor model was tested; consequently, no total score is reported. The two-item physical mistreatment subscale should be regarded as preliminary, and the empirical distinction between discrimination and sexual harassment is not yet firmly established, since merging these two dimensions produced only a negligible change in model fit. To our knowledge, this is one of the first instruments designed to assess multiple types of mistreatment among medical students and medical trainees whose factor structure has been examined through both exploratory and confirmatory analyses in independent samples. Future research should expand the content of the physical mistreatment and discrimination dimensions, examine measurement invariance across institutions and populations, establish externally validated interpretive thresholds, and replicate these findings in other Spanish-speaking populations, as well as in English-speaking samples.

## Figures and Tables

**Table 1:** Initial 28-item pool used for exploratory factor analysis.

Item	Question English/*Spanish*
1	Persistent attempts to belittle and undermine your work/ *Intentos de menospreciar o menoscabar su trabajo* *
2	Persistent unjustified criticism and monitoring of your work/*Críticas injustificadas al supervisar/evaluar su quehacer* *
3	Persistent attempts to humiliate you in front of colleagues/*Intentos de humillarle delante de sus pares* *
4	Intimidatory use of discipline, competence procedures/*Uso intimidatorio de medidas disciplinarias* *
5	Undermining your personal integrity/*Afectación de su integridad personal* *
6	Destructive innuendo and sarcasm/*Insinuaciones dañinas o sarcásticas* *
7	Verbal and non-verbal threats/*Amenazas verbales o no verbales* *
8	Making inappropriate jokes about you/*Burlas inapropiadas acerca de usted* *
9	Persistent teasing/*Burlas persistentes* *
10	Physical violence/*Violencia física (golpes, empujones, patadas, manotazos, etc.)* *
11	Violence to property/*Violencia o daño a la propiedad personal* *
12	Withholding necessary information from you/*Le impiden acceder a información necesaria para sus estudios* *
13	Freezing out/ignoring/excluding/*Le ignoran o le excluyen* *
14	Unreasonable refusal of applications for leave, training, or promotion/*Negativas injustificadas a que pueda participar de permisos, entrenamientos o promociones* *
15	Undue pressure to produce work/*Exigencia exagerada en su quehacer* *
16	Setting of impossible deadlines/*Imposición de plazos imposibles de lograr* *
17	Shifting goalposts without telling you/*Modificación de responsabilidades sin consultarle* *
18	Constant undervaluing of your efforts/*Subvaloración de su desempeño* *
19	Persistent attempts to demoralize you/*Intentos de desmoralizarle* *
20	Overload of assigned tasks without consulting you/Sobrecarga de tareas asignadas sin consultarle **
21	Removal of areas of responsibility without consultation/*Remoción de áreas de responsabilidades sin previa consulta* *
22	Discrimination on grounds of race/*Discriminación por motivos raciales* *
23	Discrimination based on illness or disability/*Discriminación por enfermedad o discapacidad* **
24	Sexual harassment/*Acoso sexual* **
25	Discrimination on grounds of gender/*Discriminación por su género* *
26	Discrimination based on sexual orientation/*Discriminación por su orientación sexual* ***
27	Unwanted/inappropriate sexual advances/*Insinuaciones sexuales indeseadas/inapropiadas* ***
28	Offensive sexual comments directed at you/*Comentarios sexuales ofensivos hacia su persona* ***

Note: *: items adapted from the original questionnaire by Quine (2002) [13]; **: items adapted from the questionnaire by Derive et al. (2017) [11]; ***: items adapted from the questionnaire by Reyes-Arellano et al. (2019) [7].

**Table 2:** Descriptive data of the variables analyzed.

Variable	n = 555
Age, mean ± SD	23.7 ± 4.6
Female sex, n (%)	355 (64.0)
Educational level − Non-clinical undergraduate − Clinical undergraduate − Interns − Residents	242 (43.6) 92 (16.6) 46 (8.3) 175 (31.5)
In a relationship, n (%)	271 (48.8)
With children, n (%)	19 (3.4)
With job, n (%)	104 (18.7)
Socioeconomic status, n (%) − Very low − Low − Medium − High − Very high	90 (16.2) 89 (16.0) 126 (22.7) 104 (18.8) 146 (26.3)

**Table 3:** Factor loadings from the exploratory factor analysis (EFA).

Item	Factor 1	Factor 2	Factor 3	Factor 4
17	0.910			
15	0.858			
20	0.840			
21	0.794			
18	0.748			
16	0.742			
19	0.605			
13	0.494			
14	0.478		0.491	
2	0.439	0.561		
4	0.401	0.543		
9		0.795		
8		0.793		
3		0.719		
6		0.697		
5		0.612		
1		0.600		
7		0.567		
11			0.937	
10			0.815	
22			0.754	
23			0.676	
12			0.594	
26			0.525	
24				0.934
27				0.932
28				0.763
25				0.565

**Table 4:** Final 16-item version of the Mistreatment Questionnaire for Medical Trainees (MQMT).

Dimension of Mistreatment	Question English/*Spanish* (Item Preserved)
**Academic mistreatment**	Undue pressure to produce work/*Exigencia exagerada en su quehacer* (ítem 15) Setting of impossible deadlines/*Imposición de plazos imposibles de lograr* (ítem 16) Shifting goalposts without telling you/*Modificación de responsabilidades sin consultarle* (ítem 17) Constant undervaluing of your efforts/*Subvaloración de su desempeño* (ítem 18)
**Psychological mistreatment**	Persistent attempts to humiliate you in front of colleagues/*Intentos de humillarle delante de sus pares* (Item 3) Destructive innuendo and sarcasm/*Insinuaciones dañinas o sarcásticas* (ítem 6) Verbal and non-verbal threats/*Amenazas verbales o no verbales* (Item 7) Making inappropriate jokes about you/*Burlas inapropiadas acerca de usted* (ítem 8)
**Physical mistreatment**	Physical violence/Violencia física (golpes, empujones, patadas, manotazos, etc.) (Item 10) Violence to property/Violencia o daño a la propiedad personal (Item 11)
**Discrimination**	Discrimination on grounds of race/*Discriminación por motivos raciales* (ítem 22) Discrimination based on illness or disability/*Discriminación por enfermedad o discapacidad* (Item 23) Discrimination based on sexual orientation/*Discriminación por su orientación* sexual (ítem 26)
**Sexual harassment**	Sexual harassment/*Acoso sexual* (ítem 24) Unwanted/inappropriate sexual advances/*Insinuaciones sexuales indeseadas/inapropiadas* (ítem 27) Offensive sexual comments directed at you/*Comentarios sexuales ofensivos hacia su persona* (ítem 28)

**Table 5:** Response distributions of the 16 retained items in the EFA subsample (n = 280).

Dimension	Item	0 (Never), n (%)	1 (Almost Never), n (%)	2 (Sometimes), n (%)	3 (Almost Always), n (%)	4 (Always), n (%)
Academic mistreatment	15	118 (42.1)	52 (18.6)	55 (19.6)	29 (10.4)	26 (9.3)
	16	143 (51.1)	62 (22.2)	34 (12.1)	23 (8.2)	18 (6.4)
	17	141 (50.4)	59 (21.1)	43 (15.3)	21 (7.5)	16 (5.7)
	18	123 (43.9)	66 (23.6)	49 (17.5)	25 (8.9)	17 (6.1)
Psychological mistreatment	3	91 (32.5)	72 (25.7)	65 (23.2)	37 (13.2)	15 (5.4)
	6	115 (41.0)	64 (22.9)	61 (21.8)	22 (7.9)	18 (6.4)
	7	159 (56.8)	60 (21.4)	33 (11.8)	16 (5.7)	12 (4.3)
	8	140 (50.0)	71 (25.4)	34 (12.1)	20 (7.1)	15 (5.4)
Physical mistreatment	10	238 (85.0)	29 (10.4)	9 (3.2)	3 (1.1)	1 (0.3)
	11	229 (81.8)	38 (13.6)	8 (2.9)	4 (1.4)	1 (0.3)
Discrimination	22	223 (79.6)	33 (11.8)	17 (6.1)	4 (1.4)	3 (1.1)
	23	229 (81.8)	29 (10.4)	11 (3.9)	7 (2.5)	4 (1.4)
	26	231 (82.5)	30 (10.7)	13 (4.6)	5 (1.8)	1 (0.4)
Sexual harassment	24	219 (78.2)	31 (11.1)	21 (7.5)	4 (1.4)	5 (1.8)
	27	215 (76.8)	38 (13.6)	17 (6.1)	6 (2.1)	4 (1.4)
	28	221 (78.9)	30 (10.7)	18 (6.4)	8 (2.9)	3 (1.1)

**Table 6:** Fit indices of the competing CFA models in the CFA subsample (n = 275).

Model	χ^2^ (df)	*p*	CFI	TLI	RMSEA [90% CI]	SRMR	AIC	BIC
**WLSMV (primary estimator)**								
M1. One factor	581.32 (104)	<0.001	0.934	0.923	0.129 [0.119–0.140]	0.123	—	—
M2. Four factors (EFA-derived)	235.77 (98)	<0.001	0.981	0.977	0.072 [0.060–0.083]	0.057	—	—
M3. Five correlated factors (final)	206.82 (94)	<0.001	0.984	0.980	0.066 [0.054–0.078]	0.049	—	—
M4. Second-order	340.84 (99)	<0.001	0.966	0.959	0.094 [0.084–0.105]	0.076	—	—
M3a. r (academic, psychological) = 1	290.32 (95)	<0.001	0.973	0.966	0.087 [0.075–0.098]	0.056	—	—
M3b. r (physical, discrimination) = 1	233.23 (95)	<0.001	0.981	0.976	0.073 [0.061–0.085]	0.051	—	—
M3c. r (discrimination, sexual harassment) = 1	217.95 (95)	<0.001	0.983	0.978	0.069 [0.057–0.081]	0.050	—	—
**MLR (sensitivity analysis)**								
M1. One factor	593.64 (104)	<0.001	0.721	0.679	0.185 [0.170–0.199]	0.115	9396.71	9512.45
M2. Four factors (EFA-derived)	241.69 (98)	<0.001	0.923	0.906	0.100 [0.084–0.116]	0.057	8679.36	8816.80
M3. Five correlated factors (final)	153.14 (94)	<0.001	0.969	0.961	0.065 [0.045–0.083]	0.048	8514.55	8666.46
M4. Second-order	209.51 (99)	<0.001	0.943	0.931	0.086 [0.069–0.102]	0.089	8603.87	8737.69
M3a. Academic and psychological merged	225.81 (98)	<0.001	0.934	0.919	0.093 [0.077–0.109]	0.067	8637.38	8774.82
M3b. Physical and discrimination merged	241.69 (98)	<0.001	0.923	0.906	0.100 [0.084–0.116]	0.057	8679.36	8816.80
M3c. Discrimination and sexual harassment merged	174.25 (98)	<0.001	0.960	0.951	0.072 [0.055–0.090]	0.050	8550.43	8687.87

Note: WLSMV: weighted least squares with mean- and variance-adjusted test statistics on polychoric correlations (primary estimator); MLR: robust maximum likelihood (sensitivity analysis). df: degrees of freedom; χ^2^: scaled χ^2^; AIC: Akaike information criterion; BIC: Bayesian information criterion; —: not available for WLSMV. M3a–M3c are models in which a pair of highly correlated factors was constrained: under WLSMV the latent correlation was fixed at 1 (Δdf = 1), whereas under MLR, where those models did not converge, the two factors were merged into a single factor (Δdf = 4); for this reason, M3b under MLR is equivalent to M2.

**Table 7:** Fully standardized factor loadings of the final five-factor model (M3) and reliability of the subscales (n = 275).

Item	Academic Mistreatment	Psychological Mistreatment	Physical Mistreatment	Discrimination	Sexual Harassment
Item 18	0.894 (0.862–0.926)				
Item 15	0.854 (0.817–0.891)				
Item 17	0.875 (0.833–0.917)				
Item 16	0.891 (0.852–0.929)				
Item 6		0.855 (0.815–0.896)			
Item 3		0.814 (0.766–0.862)			
Item 8		0.821 (0.769–0.873)			
Item 7		0.889 (0.842–0.935)			
Item 11			0.980 (0.952–0.998)		
Item 10			0.968 (0.941–0.996)		
Item 23				0.941 (0.902–0.981)	
Item 22				0.924 (0.887–0.960)	
Item 26				0.922 (0.877–0.967)	
Item 24					0.904 (0.871–0.937)
Item 27					0.930 (0.892–0.967)
Item 28					0.951 (0.914–0.987)
α/ω	0.894/0.896	0.866/0.868	0.943/0.943; SB = 0.917	0.878/0.881	0.894/0.897
Ordinal α/model-based ω	0.931/0.901	0.907/0.873	0.975/0.922	0.947/0.898	0.945/0.914

Note: Values are fully standardized factor loadings with their 95% confidence intervals (WLSMV; n = 275). α: Cronbach’s alpha; ω: McDonald’s omega; SB: Spearman–Brown coefficient (two-item subscale). Reliability coefficients were calculated in the CFA subsample (n = 275); ordinal α is based on polychoric correlations, and model-based ω was derived from the WLSMV solution.

**Table 8:** Standardized latent correlations between the factors of the final model (WLSMV; n = 275).

Factors	Academic Mistreatment	Psychological Mistreatment	Physical Mistreatment	Discrimination	Sexual Harassment
Academic mistreatment	-	0.852 [0.804–0.901]	0.614 [0.469–0.760]	0.703 [0.618–0.789]	0.667 [0.567–0.766]
Psychological mistreatment	-	-	0.775 [0.671–0.879]	0.806 [0.736–0.877]	0.749 [0.661–0.836]
Physical mistreatment	-	-	-	0.890 [0.829–0.950]	0.781 [0.691–0.871]
Discrimination	-	-	-	-	0.943 [0.911–0.975]

Note: Standardized latent correlations estimated with WLSMV [95% confidence interval]. All correlations were statistically significant (*p* < 0.001).

**Table 9:** Distribution of participants across descriptive frequency bands for each MQMT dimension (N = 555).

Type of Mistreatment	Infrequent (Mean < 1), n (%)	Occasional (1 ≤ Mean < 2), n (%)	Recurrent (Mean ≥ 2), n (%)
Academic	290 (52.3)	141 (25.4)	124 (22.3)
Psychological	298 (53.7)	166 (29.9)	91 (16.4)
Physical	471 (84.8)	62 (11.2)	22 (4.0)
Discrimination	466 (84.0)	66 (11.9)	23 (4.1)
Sexual	454 (81.8)	62 (11.2)	39 (7.0)

Note: Frequency bands are anchored to the response labels and are descriptive categories developed for this study; they are not validated cut-off points.

**Table 10:** Correlations between MQMT scores and indicators of psychological discomfort.

Scale	Academic Mistreatment	Psychological Mistreatment	Physical Mistreatment	Discrimination	Sexual Harassment
GAD-7	0.357	0.326	0.136	0.175	0.172
PHQ-9	0.349	0.344	0.134	0.221	0.193
PSS-10	0.370	0.313	0.085	0.126	0.150

Note: Spearman’s correlation coefficients (ρ) in the total sample (N = 555). All coefficients were statistically significant (*p* < 0.05).

## Data Availability

The datasets generated and analyzed during the current study are available from the corresponding author upon reasonable request due to ethical restrictions and privacy of the personal data of the participants.

## Supplementary Materials

The following supporting information can be downloaded at: https://www.mdpi.com/article/10.3390/healthcare14193333/s1, Table S1: Item-level psychometric properties and retention decisions for the initial 28-item pool (intended domain, EFA loadings, communalities, decision, reason, and stage of decision). Table S2: Fit indices for the competing factor models; Table S3: Nested model comparisons in the CFA subsample with the WLSMV estimator; Table S4: Nested model comparisons in the CFA subsample with the MLR estimator.
