# Supplementary material for: Development and Psychometric Validation of the Mistreatment Questionnaire for Medical Trainees (MQMT) in Mexican Medical Students, Interns, and Residents
Source: Healthcare (Basel). 2026 Oct 6;14(19):3333. doi: 10.3390/healthcare14193333 (PMC13665546; doi:10.3390/healthcare14193333)
Supplement: Supplementary file 1 [file healthcare-14-03333-s001.zip › healthcare-4569852-supplementary.pdf]

Supplementary Material

Supplementary Table S1. Item-level psychometric properties and retention decisions for the initial 28-item pool.

Item	Original item	Theoretical domain	F1	F2	F3	F4	Communality	Loading difference	Retained	Reason	Elimination phase
1	WBJDQ_1	Psychological	0.378	0.600	-0.199	0.025	0.697	0.222	No	Semantic redundancy with another item in the same factor that has a higher factor loading.	Pre-CFA
2	WBJDQ_2	Psychological	0.439	0.561	-0.158	0.002	0.736	0.122	No	Cross-loading	Pre-CFA
3	WBJDQ_3	Psychological	0.297	0.719	-0.201	0.026	0.773	0.422	Yes	High factor loading and theoretical relevance	Retained

4	WBJDQ_4	Psychological	0.401	0.543	-0.108	0.046	0.722	0.142	No	Cross-loading	Pre-CFA
5	WBJDQ_5	Psychological	0.100	0.612	0.072	0.121	0.637	0.491	No	Semantic redundancy with another item in the same factor that has a higher factor loading or greater theoretical relevance	Pre-CFA
6	WBJDQ_6	Psychological	0.085	0.697	0.009	0.036	0.616	0.612	Yes	High factor loading, theoretical relevance	Retained
7	WBJDQ_7	Psychological	0.181	0.567	0.206	-0.041	0.673	0.361	Yes	Theoretical relevance and good	Retained

										factor loading	
8	WBJDQ_8	Psychological	-0.093	0.793	0.203	-0.061	0.661	0.590	Yes	High factor loading, theoretical relevance	Retained
9	WBJDQ_9	Psychological	-0.082	0.795	0.286	-0.068	0.767	0.509	No	Semantic redundancy with another item in the same factor that has greater theoretical relevance	Pre-CFA
10	WBJDQ_10	Physical	-0.194	0.028	0.815	0.150	0.684	0.621	Yes	Essential for content coverage	Retained
11	WBJDQ_11	Physical	-0.232	0.130	0.937	0.022	0.817	0.705	Yes	Essential for content coverage	Retained

12	WBJDQ_12	Academic	0.274	-0.018	0.594	-0.069	0.529	0.320	No	Loading on a factor inconsistent with the conceptual content of the item	Pre-CFA
13	WBJDQ_13	Psychological	0.494	0.195	0.161	0.030	0.592	0.299	No	Semantic redundancy with another item in the same factor that has a higher factor loading or greater theoretical relevance	Pre-CFA
14	WBJDQ_14	Academic	0.478	0.034	0.491	-0.109	0.670	0.013	No	Cross-loading	Pre-CFA

15	WBJDQ_15	Academic	0.858	0.089	-0.111	-0.028	0.741	0.747	Yes	High factor loading, theoretical relevance	Retained
16	WBJDQ_16	Academic	0.742	0.087	-0.071	0.130	0.694	0.612	Yes	High factor loading, theoretical relevance	Retained
17	WBJDQ_17	Academic	0.910	-0.038	0.012	-0.003	0.787	0.872	Yes	High factor loading, theoretical relevance	Retained
18	WBJDQ_18	Academic	0.748	0.137	-0.057	0.038	0.708	0.611	Yes	High factor loading, theoretical relevance	Retained
19	WBJDQ_19	Psychological	0.605	0.254	0.085	-0.074	0.688	0.351	No	Semantic redundancy with another item in	Pre-CFA

										the same factor that has a higher factor loading or greater theoretical relevance	
20	Derive_Academic_2	Academic	0.840	-0.022	0.004	-0.026	0.666	0.814	No	Semantic redundancy with another item in the same factor that has greater theoretical relevance	Pre-CFA
21	WBJDQ_20	Academic	0.794	-0.146	0.279	-0.071	0.698	0.515	No	Semantic redundancy with another item in the same factor that has	Pre-CFA

										greater theoretical relevance	
22	WBJDQ_21	Discrimination	-0.002	0.097	0.754	-0.006	0.645	0.657	Yes	High factor loading, theoretical relevance	Retained
23	Derive_Discrimination_8	Discrimination	0.114	0.041	0.676	-0.057	0.543	0.562	Yes	High factor loading, theoretical relevance	Retained
24	Derive_Sexual_7	Sexual	0.018	-0.061	0.026	0.934	0.861	0.873	Yes	High factor loading, theoretical relevance	Retained
25	WBJDQ_21	Discrimination	0.250	0.037	-0.019	0.565	0.516	0.315	No	Loading on a factor inconsistent with the conceptu	Pre-CFA

										al content of the item	
26	Reyes_18	Discrimination	0.132	-0.189	0.525	0.209	0.412	0.316	Yes	Essential for content coverage	Retained
27	Reyes_14	Sexual	-0.038	-0.023	0.028	0.932	0.850	0.894	Yes	High factor loading, theoretical relevance	Retained
28	Reyes_15	Sexual	-0.173	0.144	0.191	0.763	0.776	0.572	Yes	High factor loading, theoretical relevance	Retained

Note: EFA subsample (n = 280); unweighted least squares extraction with Promax rotation on Pearson correlation matrix. F1 = academic mistreatment factor; F2 = psychological mistreatment factor; F3 = physical mistreatment/discrimination factor; F4 = sexual harassment factor. Δ loading = difference between the primary and the highest secondary loading; values < 0.15 were considered cross-loadings. Theoretical domain = domain the item was intended to represent when the item pool was assembled. Pre-CFA = decision made in the EFA subsample before the confirmatory model was specified. Items retained in the final 16-item MQMT: 3, 6, 7, 8, 10, 11, 15, 16, 17, 18, 22, 23, 24, 26, 27, and 28.

Supplementary Table S2. Fit indices for the competing factor models

Estimator / Model	χ^2_scaled	df	p	CFI	TLI	RMSEA [90% CI]	SRMR	AIC	BIC
**WLSMV (primary)**									
M1 (1 factor)	581.32	104	< .001	.934	.923	.129 [.119, .140]	.123	—	—
M2 (4 factors)	235.77	98	< .001	.981	.977	.072 [.060, .083]	.057	—	—
M3 (5 factors)	206.82	94	< .001	.984	.980	.066 [.054, .078]	.049	—	—
M4 (second-order)	340.84	99	< .001	.966	.959	.094 [.084, .105]	.076	—	—
M3 in EFA sample	189.92	94	< .001	.990	.988	.060 [.048, .073]	.053	—	—
**MLR (sensitivity)**									
M1 (1 factor)	593.64	104	< .001	.721	.679	.185 [.170, .199]	.115	9396.7	9512.4
M2 (4 factors)	241.69	98	< .001	.923	.906	.100 [.084, .116]	.057	8679.4	8816.8

M3 (5 factors)	153.14	94	< .001	.969	.961	.065 [.045, .083]	.048	8514.6	8666.5
M4 (second-order)	209.51	99	< .001	.943	.931	.086 [.069, .102]	.089	8603.9	8737.7

Note: M1 = unidimensional model (16 items loading on a single general factor); M2 = four-factor model derived from the exploratory factor analysis (physical mistreatment and discrimination merged into a single factor); M3 = five-factor correlated model (final model); M4 = second-order model with a general mistreatment factor (GEN) underlying the five specific dimensions. WLSMV = weighted least squares with mean- and variance-adjusted test statistics on the matrix of polychoric correlations; MLR = robust maximum likelihood. χ^2_scaled = scaled chi-square; df = degrees of freedom; CFI = comparative fit index; TLI = Tucker-Lewis index; RMSEA = root mean square error of approximation with 90% confidence interval; SRMR = standardized root mean square residual; AIC = Akaike information criterion; BIC = Bayesian information criterion. AIC and BIC are not reported for WLSMV because this estimator does not provide likelihood-based information criteria. The M3 model was also fitted in the EFA subsample (n = 280) as a cross-validation check; this row is not directly comparable with the CFA rows because it was estimated on a different sample. All models were estimated in the CFA subsample (n = 275) unless otherwise noted.

Supplementary Table S3. Nested model comparisons in the CFA subsample with WLSMV estimator

**Comparison**	**Scaled Δχ^2**	**Δdf**	**p**	**ΔCFI**	**ΔRMSEA**	**ΔSRMR**
**WLSMV Estimator**						
M2 (4 factors) vs. M3 (5 factors)	22.48	4	< .001	−.003	0.005	0.008
M4 (second-order) vs. M3	66.70	5	< .001	−.018	0.028	0.027
M1 (1 factor) vs. M3	176.03	10	< .001	−.051	0.063	0.074
M3 with r(PHY, DIS) = 1 vs. M3	13.71	1	< .001	−.004	0.007	0.002
M3 with r(ACA, PSY) = 1 vs. M3	41.14	1	< .001	−.011	0.020	0.007
M3 with r(DIS, SEX) = 1 vs. M3	13.41	1	< .001	−.001	0.003	0.001

Note: Scaled Δχ^2 = scaled-and-shifted difference test for WLSMV. ΔCFI, ΔRMSEA, and ΔSRMR = value of the compared model minus that of M3. For CFI, negative values indicate that the constrained model fits worse than M3; for RMSEA and SRMR, positive values indicate worse fit. ACA = academic mistreatment; PSY = psychological mistreatment; PHY = physical mistreatment; DIS = discrimination; SEX = sexual harassment.

Supplementary Table S4. Nested model comparisons in the CFA subsample with MLR estimator

**Comparison**	**Scaled Δχ^2**	**Δdf**	**p**	**ΔCFI**	**ΔRMSEA**	**ΔSRMR**	**ΔAIC**	**ΔBIC**

**MLR Estimator**								
M2 (4 factors) vs. M3 (5 factors)	58.10	4	< .001	−.046	0.035	0.009	164.81	150.34
M4 (second-order) vs. M3	66.32	5	< .001	−.026	0.021	0.042	89.32	71.24
M1 (1 factor) vs. M3	253.61	10	< .001	−.248	0.120	0.067	882.16	845.99

Note: Scaled $\Delta\chi^2$ Satorra–Bentler scaled difference test for MLR. ΔCFI, ΔRMSEA, and ΔSRMR = value of the compared model minus that of M3. For CFI, negative values indicate that the constrained model fits worse than M3; for RMSEA and SRMR, positive values indicate worse fit. Positive ΔAIC and ΔBIC indicate a worse balance between fit and parsimony than M3.
